# Structural variability and complexity of the giant *Pithovirus sibericum* particle revealed by high-voltage electron cryo-tomography and energy-filtered electron cryo-microscopy

**DOI:** 10.1038/s41598-017-13390-4

**Published:** 2017-10-16

**Authors:** Kenta Okamoto, Naoyuki Miyazaki, Chihong Song, Filipe R. N. C. Maia, Hemanth K. N. Reddy, Chantal Abergel, Jean-Michel Claverie, Janos Hajdu, Martin Svenda, Kazuyoshi Murata

**Affiliations:** 1 Laboratory of Molecular Biophysics, Department of Cell and Molecular Biology, Uppsala University, Husargatan 3 (Box 596), SE-75124 Uppsala, Sweden; 2 National Institute for Physiological Sciences (NIPS), Okazaki, Aichi 444-8585 Japan; 3 Structural and Genomic Information Laboratory, UMR 7256 (IMM FR 3479) Centre National de la Recherche Scientifique & Aix-Marseille University, Marseille, 13288 France; 4 Assistance Publique des Hôpitaux de Marseille. La Timone, 13005 Marseille, France; 5 Institute of Physics AS CR, v.v.i., Na Slovance 2, 18221 Prague 8, Czech Republic

## Abstract

The *Pithoviridae* giant virus family exhibits the largest viral particle known so far, a prolate spheroid up to 2.5 μm in length and 0.9 μm in diameter. These particles show significant variations in size. Little is known about the structure of the intact virion due to technical limitations with conventional electron cryo-microscopy (cryo-EM) when imaging thick specimens. Here we present the intact structure of the giant *Pithovirus sibericum* particle at near native conditions using high-voltage electron cryo-tomography (cryo-ET) and energy-filtered cryo-EM. We detected a previously undescribed low-density outer layer covering the tegument and a periodical structuring of the fibres in the striated apical cork. Energy-filtered Zernike phase-contrast cryo-EM images show distinct substructures inside the particles, implicating an internal compartmentalisation. The density of the interior volume of Pithovirus particles is three quarters lower than that of the Mimivirus. However, it is remarkably high given that the 600 kbp Pithovirus genome is only half the size of the Mimivirus genome and is packaged in a volume up to 100 times larger. These observations suggest that the interior is densely packed with macromolecules in addition to the genomic nucleic acid.

## Introduction

Following the discovery of the giant Mimivirus in 2003^1^, numerous virus species with viral particles sizes exceeding 500 nm in diameter, have been identified from aquatic and terrestrial samples^2^. Some of these viruses have well defined capsid structures with pseudo icosahedral symmetry, like Mimivirus^1,3^, while other even larger viruses exhibit non-icosahedral capsids. Representative examples of the giant non-icosahedral viruses are *Pandoravirus salinus*, isolated off the Chilean coast, *Pandoravirus dulcis* isolated from a fresh water pond in Australia, and *Pandoravirus inopinatum* isolated from a pair of contact lenses^4–6^. So far, the largest viral particles are exhibited by the *Pithoviridae* family^7–9^. *Pithovirus sibericum* was isolated from a 30,000 year-old layer of the Siberian permafrost^7^ and the shape of its particle resembles a “pithos”, a large amphora used by the ancient Greeks. The giant virus *Mollivirus sibericum* was also isolated from the same sample, but is phylogenetically unrelated to Pithoviruses^10^. Its particles are nearly spherical, 0.6 μm in diameter.

In this study, we compared the well-characterized virion structure of Mimivirus with that of *Pithovirus sibericum*. The Mimivirus particle has a pseudo-icosahedral capsid of 0.45 µm in diameter with a starfish-like structure called “stargate” on one of the 5-fold vertices^11^. The capsid is surrounded by a uniform layer of 150 nm long peptidoglycan fibres protruding from the surface^3,12–16^. Conventional EM images of heavy metal-stained, ultrathin sections of the Mimivirus particles show a multi-layered membrane structure lining the inside of the Mimivirus capsid^14,17^. A study using the high penetration power of X-rays indicated a complex layout of the interior^18^. The surface of the Pithovirus particles is significantly different from that of the Mimivirus particles in that the virions are covered with a thick electron dense tegument made up of stripes perpendicular to the virion surface in heavy metal-stained ultrathin sections^7^.

In contrast to the uniform capsid structure of the Mimivirus particles, the particles of the Pithovirus have relatively flexible structures^2^. During the formation of the virions, the particles are first built as cylinders. Their shape is then subsequently modified into their mature form before their release from the dying amoeba cell^7^. The mature virus particles have a capped pore at one end of the ovoid, giving it a shape reminiscent of an amphora (“pithos”). In other less frequent cases, both extremities of the ovoid exhibit the same pore/cap structure^9^. The pore is capped by a striated cork-like structure, presenting a honeycomb-like framework. Currently, there is no high-resolution structure for the entire virion of the Pithovirus at near native conditions, owing to the large particle size.

Giant amoeba viruses, including Pithovirus, are not only unique in their sizes and shapes, but also characteristic in their genomes, which are much larger than most other viral genomes. About two-thirds of their predicted genes correspond to open reading frames without recognizable database homologs, and one-third encode proteins with relatives in the amoebal hosts, other eukaryota, bacteria, archaea and other large DNA viruses^2^. Their large double-stranded (ds)DNA genome is packed into the viral nucleoid core during virion assembly^4,11,17^. There is no correlation between their particle sizes and genome sizes, where Mimivirus (1.2 Mbp genome is packed in the 0.45 μm diameter particle)^3,19,20^, Mollivirus (0.6–0.7 Mbp genome is packed in the 0.6 µm diameter particle)^10^, Pandoravirus (1.9–2.5 Mbp genome is packed in the 1 µm length ovoid particle)^4^, and Pithovirus (0.6–0.7 Mbp genome is packed in the ~1.5 µm length ovoid particle)^7–9^. It is unclear how the dsDNA genomes are packaged and organized within the diverse giant virus particles.

In electron cryo-microscopy (cryo-EM) experiments, the sample is embedded in a thin vitreous ice film and we observe the projection images. Multiple and inelastic scattering of electrons in thick samples, like intact Pithovirus particles, pose a problem in regular cryo-EM studies. The inelastic mean free path of an electron at an acceleration voltage of 120 kV is about 200 nm in ice^21^. At an accelerating voltage of 200 kV, the mean free path will be approximately 1.3 (=(200k/120k)^1/2^) times longer than that at 120 kV^22^. The thickness of the Pithovirus particle is around 800 nm. With increasing sample thickness, inelastic scattering and electron cascades become dominant and lead to enhanced background noise in a bright field image^23^. From a practical perspective, the sample thickness must be less than about two to three hundred nanometers at 200 kV accelerating voltage. Energy filtering with a magnetic prism and an energy slit^22^ can be used to remove specimen-derived inelastically scattered electrons, thereby creating improved images with better contrast with zero energy loss electrons. The Zernike phase plate is made by a thin carbon film that shifts the phase of the scattered electron beam by π/2, while the unscattered background electron beam pass through the central hole of the plate^24,25^. The contrast of the image is dramatically improved by the phase-shift, converting the low-frequency sine CTF function to a cosine function in the focus^25,26^. The Zernike phase plate for TEM is also helpful in observing internal structures of a cell like a thick bacterial cell^27,28^. The recently invented hole-free phase plate, which is a derivative of the Zernike phase plate, reduces the delocalization in the images as compared to Zernike phase plate^29–31^. Electron cryo-tomography (cryo-ET)^32^ is particularly sensitive to sample thickness because the effective thickness of the vitreous ice film, embedding the specimens, increases with increased tilt angles (the electron paths at 30°, 45°, 60° tilt angle are 1.15, 1.41, and 2.00 times longer than at 0° tilt angle). Tomography using a scanning electron transmission microscopy (STEM) has advantages in reducing chromatic aberration and inelastic scattering in the thick sample^33^. STEM cryo-ET reconstructions of thick cellular specimens were also described previously^34–36^. However, since the resolution is compromised for thick samples with the currently available cryo-STEMs, especially due to multiple elastic scattering^34^, high-voltage transmission electron microscopy was primarily chosen for this study.

In the present study, we used a high-voltage cryo-EM (cryo-HVEM) at 1 MV and also an energy-filtered cryo-EM at 200 kV equipped with a Zernike phase plate to investigate the whole and fine structures of the intact giant virus particles. High-voltage electron microscopes were specifically developed for imaging relatively thick samples such as bacteria^37,38^, yeast^23^ and neuronal cells^39,40^. The higher acceleration voltage of these microscopes give a longer electron mean-free path in the sample, and this was exploited in the present study to obtain 3D tomograms of the Pithovirus particles in a cryogenic state. In contrast, the energy-filtered 200 kV electron microscope with the Zernike phase plate was used to study some of the peripheral and internal structures of the intact virus particles. Given its aforementioned advantage in studying internal structures of thick samples^27^, the Zernike phase contrast cryo-EM was used to identify interior membranous structures with the strong fringes next to densities with high intensity. Combining results from two types of microscopes provide new insights into the giant Pithovirus structure.

## Results

### Variability in size and shape

**Figure 1:**
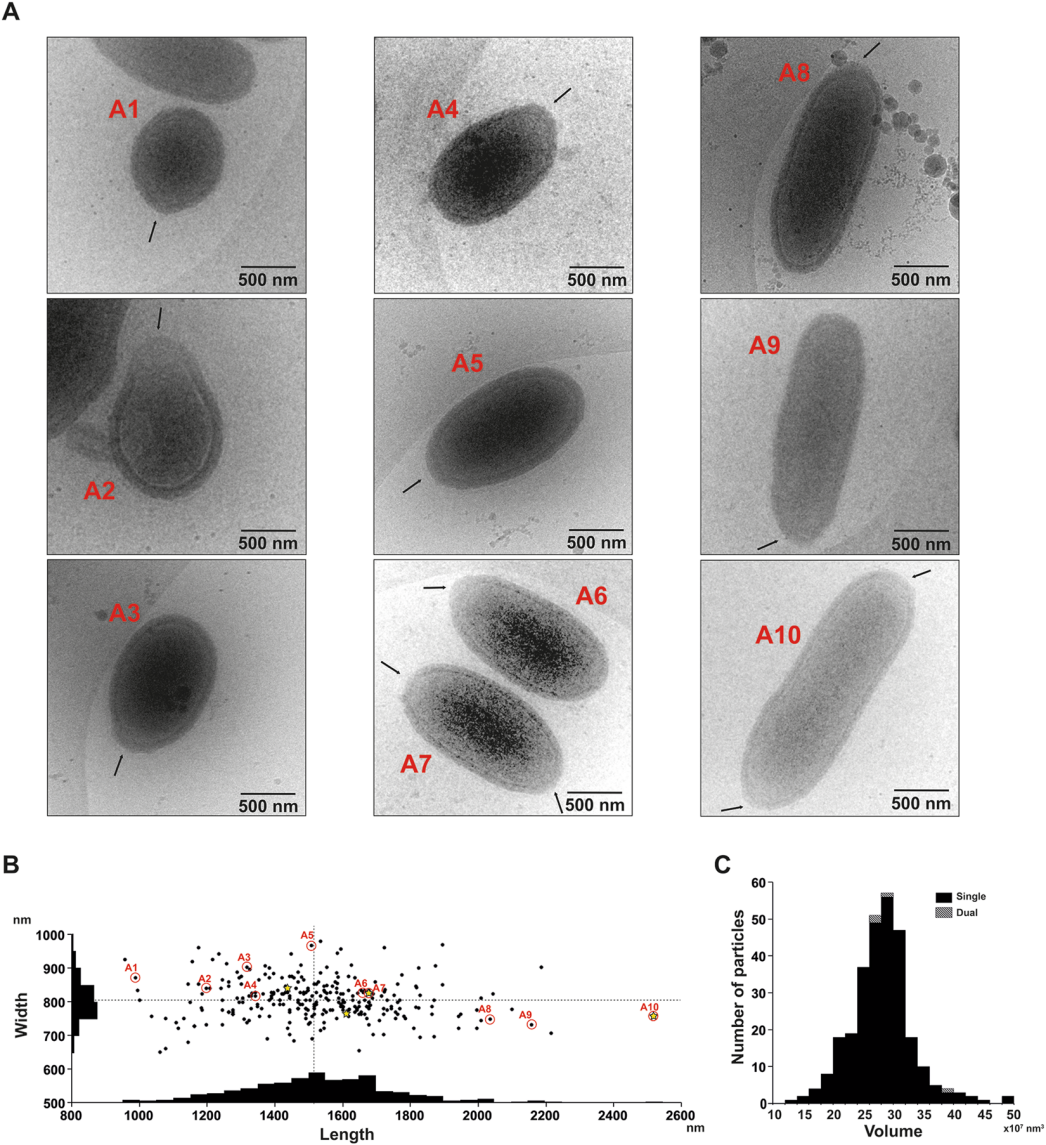
Image gallery of ice-embedded Pithovirus particles using 1 MV cryo-HVEM. The small, medium, and large sized viruses are indicated in (A1–10). The arrows indicate the positions of apical pores in the particles. (**B**) Size distribution of the Pithovirus particles, showing histograms of the length and width on each axis. The dotted lines mark the mean values for the length and the width of Pithovirus particles. Dimensions of particles shown in Figures 1–10 are marked with red circles in the histogram in B. The particles with dual pores are marked with yellow stars. (**C**) The size distribution in volume of the Pithovirus particles with single pore or dual pores.

**Figure 2:**
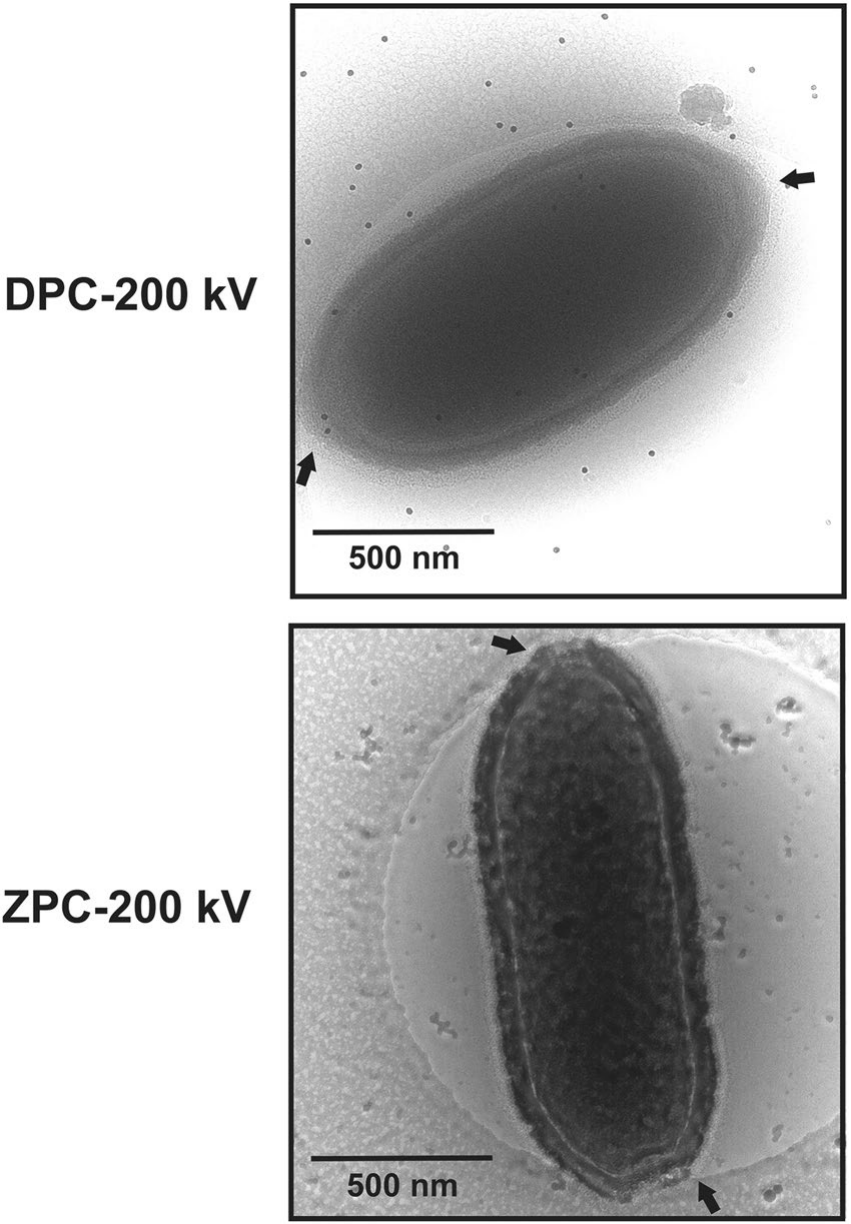
Pithovirus particles with dual corks in energy-filtered regular defocus phase contrast (DPC) (**A**) and Zernike phase contrast (ZPC) cryo-EM images (**B**) at 200 kV. Black arrows indicate the positions of the apical corks. Dark spots in A show fiducial markers of gold colloids.

Figure 1 shows Pithovirus particles embedded in vitreous ice directly imaged by cryo-HVEM at 1 MV accelerating voltage. The apical pore is highlighted by arrows, indicating that the oblong viruses were packed lying sideways in the thin ice layer. The virus particles exhibit varying sizes in the cryo-EM images. The size variation of the lying down Pithovirus particles was measured in length (major axis of the particle) and width (minor axis of the particle). The most frequently observed particles ranged between 1,350 and 1,650 nm in length and between 750 and 850 nm in width (Fig. 1B). The smallest and the largest, rarely observed, particles recorded were ~900 nm and ~2,500 nm in length and ~600 nm and ~900 nm in width (Fig. 1 A 1 and A3). Figure 1B shows a histogram of the lengths and the widths of all particles, with mean values of 1,530 ± 230 nm and 810 ± 60 nm, respectively. The variation of the width is about four times smaller than that of the length. The largest virus particles imaged in this study were a couple of hundred nanometres larger than the maximum length of 1.5 µm previously reported from chemically fixed and sectioned samples. Some of the particles showed apical pores at both poles (Fig. 1 A 7,A10), which was also confirmed by energy-filtered and Zernike phase-contrast cryo-EM studies at 200 kV (Fig. 2). This infrequent feature was only observed in relatively long particles (Fig. 1B). The volumes of the Pithovirus particles were calculated by their shapes approximated to be an ellipsoid with the length as a major axis and the width as a diameter of the minor axis (Fig. 1C). The mean volume value is 26.2 ± 5.1 × 10^7^ nm^3^ in the particles with a single pore (Fig. 1C).

### Peripheral ultrastructures of the Pithovirus particle

**Figure 3:**
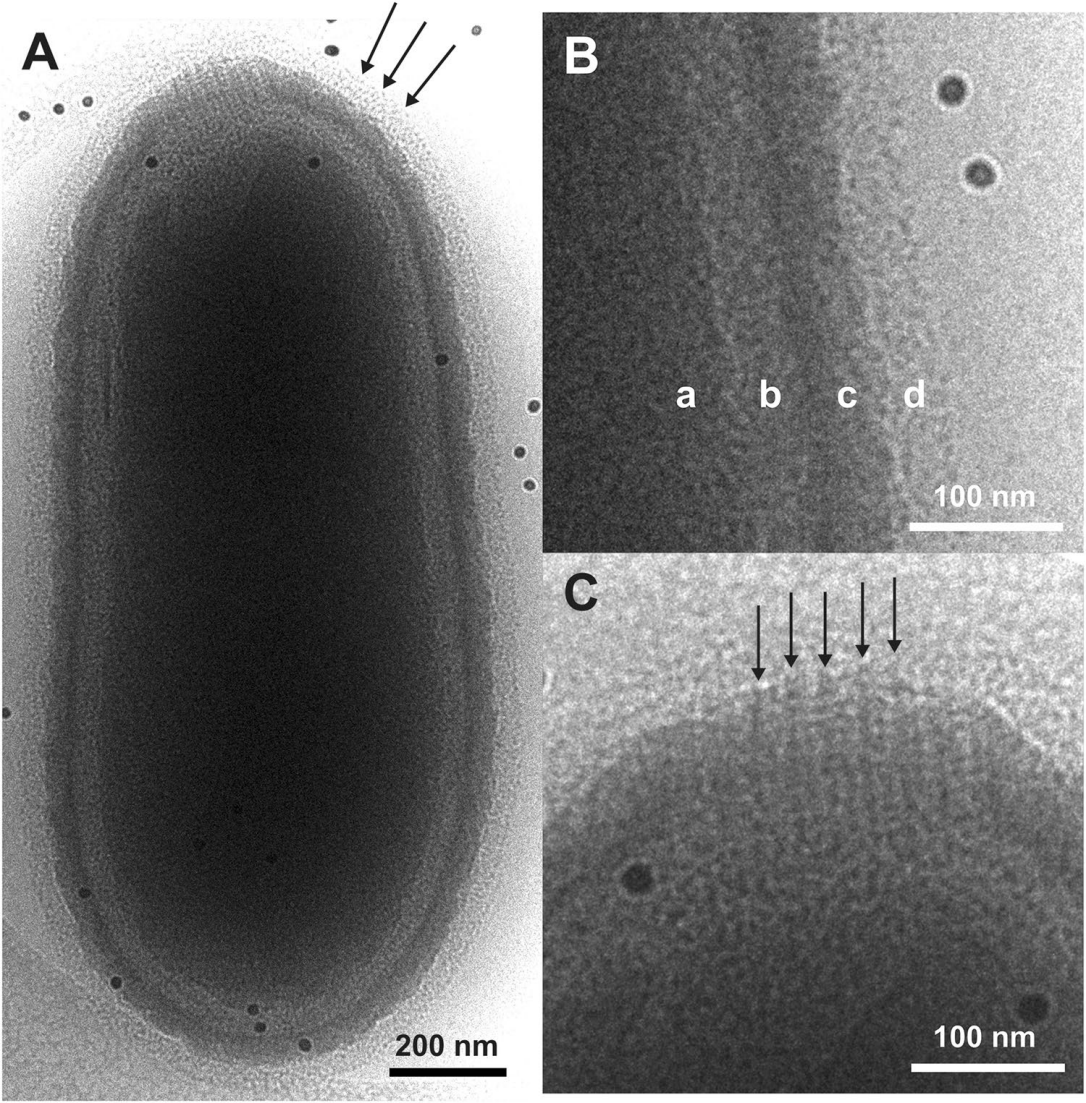
Peripheral structures of the Pithovirus particle imaged using energy-filtered cryo-EM at 200 kV. (**A**) The entire structure of the Pithovirus particle. Arrows indicate the low-density outer layer identified in this study. (**B**) Close-up view of the viral surface. The surface of the Pithovirus particle consists of four layers, those are, (a) nucleoid, (b) interior gap, (c) tegument and (d) low-density outer layer. (**C**) Close-up view of the apical cork. Arrows indicate vertical stripes that are formed at intersections of the horizontal parallel lines. Dark dots show fiducial markers of gold colloids.

Figure 3A shows a whole Pithovirus particle visualized by energy-filtered cryo-EM at 200 kV. The internal nucleoid of the virion (label **a** in Fig. 3B) is enclosed within a putative membrane layer (shown between labels **a** and **b** in Fig. 3B). This layer is further covered with a thick-layered tegument (label **c** in Fig. 3B). An irregular interior gap can be observed between the putative membrane and the tegument (label **b** in Fig. 3B). The outermost surface layer consists of a low-density material that covers the entire surface of the virus particle, including the apical pore (Arrows in Fig. 3A). This low-density outer layer was not visible in chemically fixed, dehydrated virus particles embedded in plastic and then sectioned^7^. In our observation, the low-density outer layer of about 40 nm in thickness is a newly identified layer located outside of the high-density tegument (**d** in Fig. 3B).

**Figure 4:**
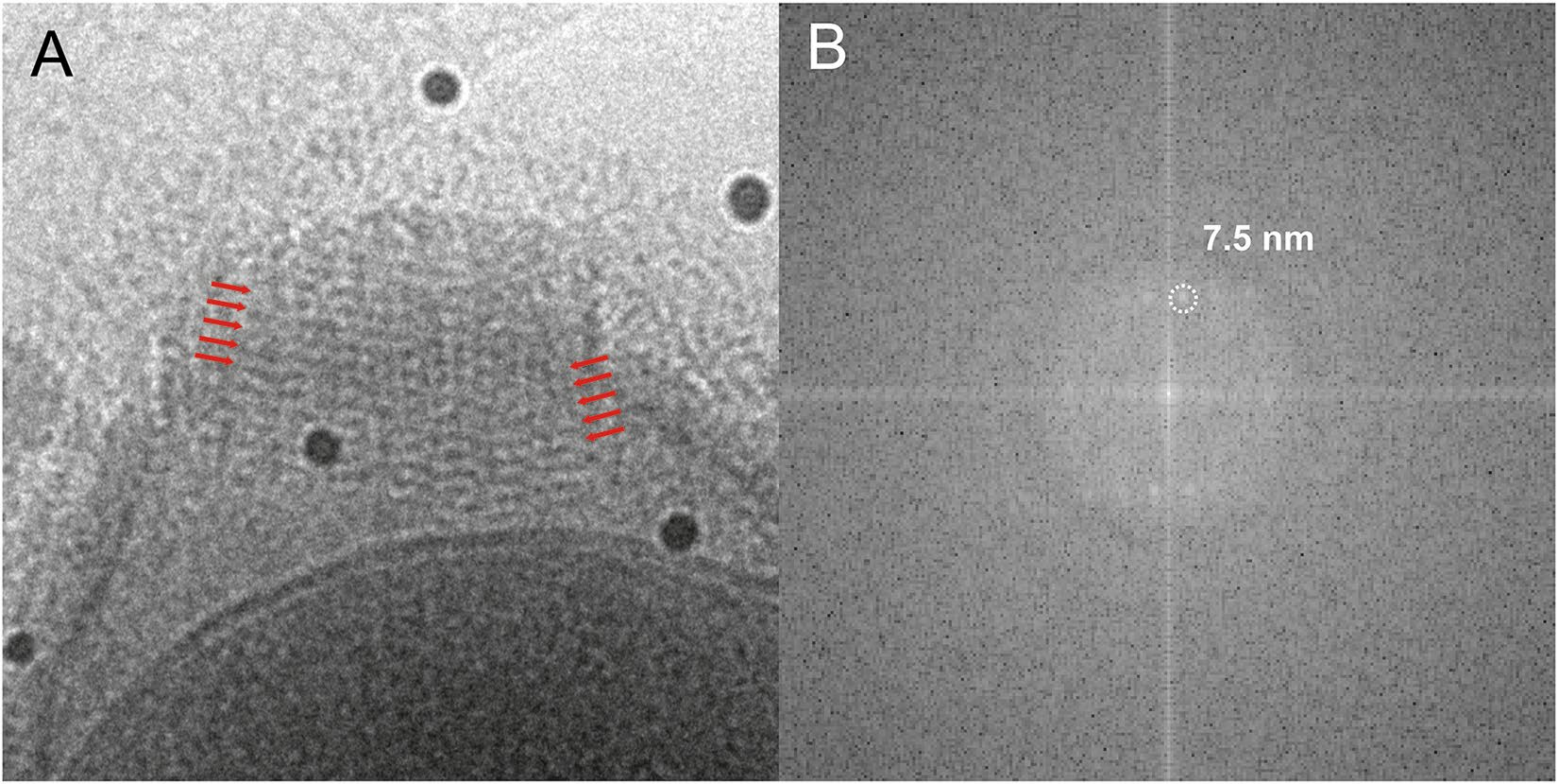
Periodic Moiré pattern of the parallelly aligned fibre structures of the apical cork in a side view of the Pithovirus particle using energy-filtered cryo-EM at 200 kV. (**A**) Close-up view of the Moiré pattern from the fibre structures of the apical cork. Red arrows indicate two sets of parallel lines in the apical cork. (**B**) Fourier transform of (**A**).

The virion has a striated cork with a honeycomb framework that caps the apical pore, a structure that was previously observed in thin-sections of plastic-embedded particles^7^. The cork was shown to be made up of 15-nm wide vertical stripes when viewing the virus particle perpendicular to its long axis (side view). The energy-filtered cryo-EM images here also show the vertical stripes in the apical cork (arrows in Fig. 3C). We further identify an additional horizontal Moiré pattern on the stripes in the side view (red marks in Fig. 4A). The pattern shows a periodicity with a pitch of approximately 7.5 nm in the particles lying sideways as determined by a Fourier transformation of the image (Fig. 4B, Supplementary Fig. S1).

### Tomographic 3D imaging of the entire Pithovirus particles

**Figure 5:**
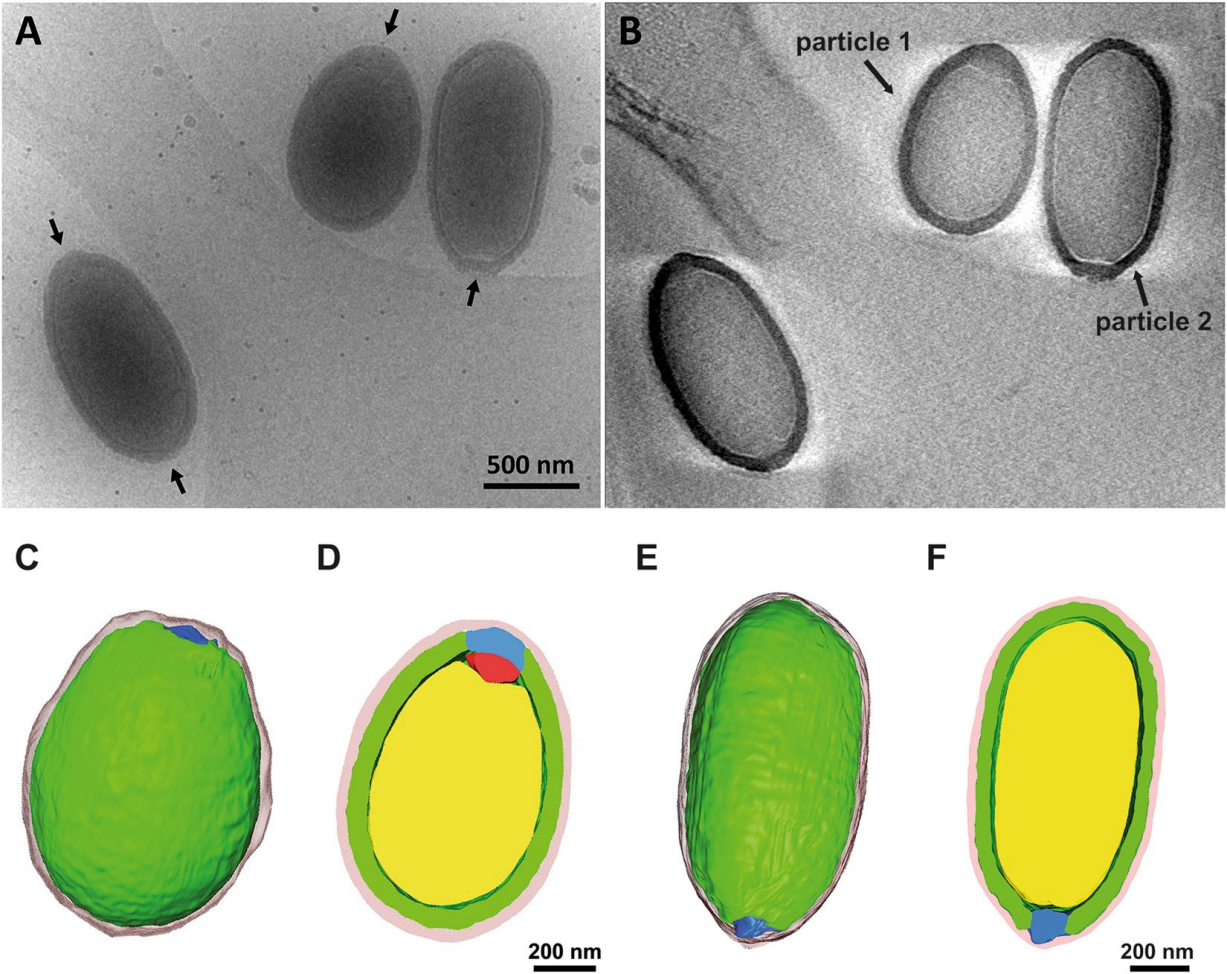
Tomographic 3D reconstructions of the Pithovirus particles using cryo-HVEM at 1 MV. (**A**) Non-tilted image of ice-embedded Pithovirus particles. Arrows indicate the positions of the apical pores. (**B**) A Z-slice of the tomographic 3D reconstruction. (**C**–**F**) Structural segmentations of the entire 3D structures of the individual Pithovirus particles. Pithovirus particles with small length (**C**–**D**, particle 1), and medium length (**E**–**F**, particle 2). (**C** and **E**) Outer surface image of the segmentations. (**D** and **F**) Cross-section images of the segmentations. In the segmentation images, yellow, green, red, blue and pink surfaces indicate nucleoid, tegument, a root of the apical cork, apical cork, and low-density outer layer, respectively.

**Figure 6:**
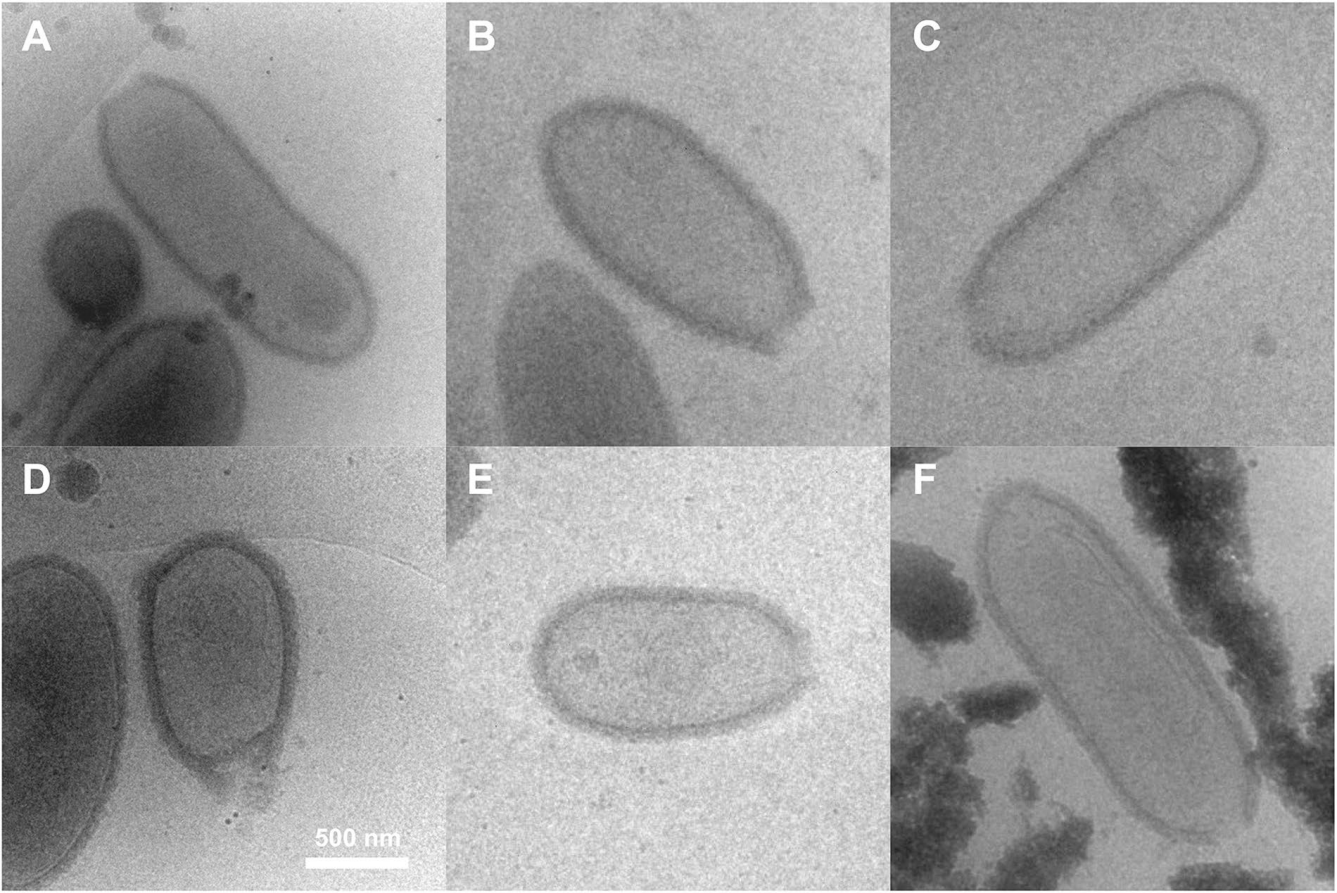
Empty Pithovirus particles imaged by cryo-HVEM at 1 MV. Membranous and other densely organized structures are seen inside the empty particles.

**Figure 7:**
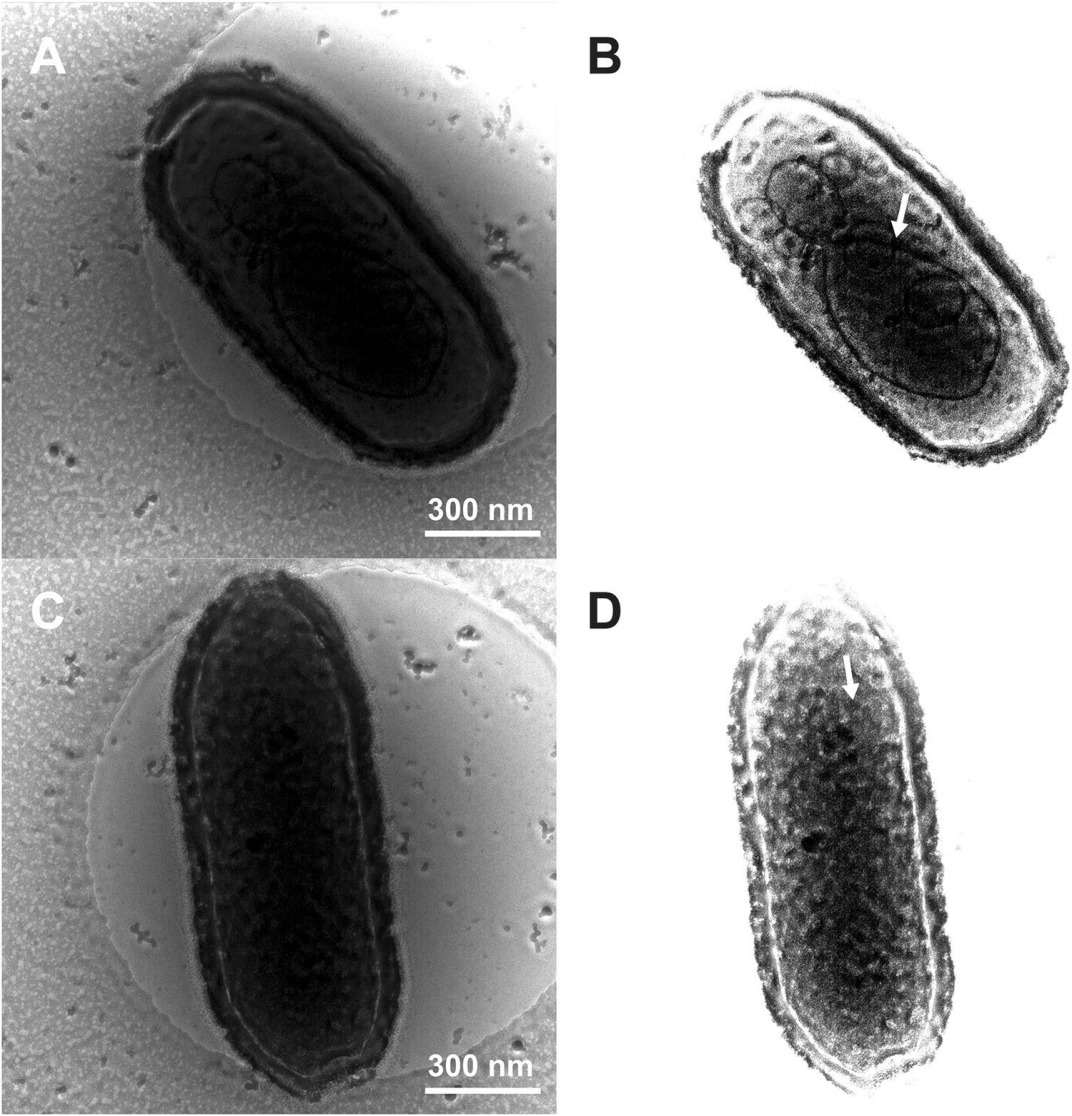
Energy-filtered Zernike phase contrast cryo-EM images of the dsDNA-full Pithovirus particles at 200 kV. (**A**,**C**) Original raw images and (**B**,**D**) raw images with higher contrast level. White arrows indicate putative interior substructures of the Pithovirus particles.

Figure 5A,B and supplementary movies S1, S2, S3 show the first 3D tomographic reconstructions of the entire Pithovirus particles in a near native state. These studies were carried out with a cryo-HVEM at 1 MV. Electrons accelerated by 1 MV in the microscope were able to penetrate the whole volume and give enough contrast in the thick specimen embedded in ice (Fig. 5A). No energy filter was used in these studies. The Pithovirus particles were segmented in the 3D tomogram and their peripheral and interior features were annotated (Fig. 5 C– 5F, Movie S3). In the 3D tomograms the root of the cork is sometimes occupied by a non-striated dense material (red region in Fig. 5D). This coincides with an indention of the nucleoid in this area. A previous study^7^ described a folded membrane structure under the cork that could be expelled together with the cork to allow fusion with the host vacuole membrane during infection. The membrane is likely linked to the cork and becomes folded only in the particles. In some particles, the non-striated material is not visible, and the cork may be more directly associated with the nucleoid membrane (Fig. 5F). The relative intensity of the differently sized Pithovirus particles in the tomograms is shown in Supplementary Fig. S2. The relative intensity of the nucleoid layer is 0.73–0.81 times lower than that of the tegument layer. The relative intensity of the nucleoid does not fluctuate noticeably across the particles (Supplementary Fig. S2). The relative nucleoid intensity is not remarkably different among the three particles with different sizes (Supplementary Fig. S2). The 3D tomogram of the Mimivirus particles is shown in Supplementary Fig. S3. The averaged relative intensity of the nucleoid layer of Mimivirus is 0.90 times lower than that of the capsid layer. Empty Pithovirus particles show membranous compartments inside the particles (Fig. 6). Similar substructures are also visible in intact Pithovirus particles in Zernike phase-contrast images with energy filtering (Fig. 7).

### Comparison of interior densities between Pithovirus and Mimivirus

**Figure 8:**
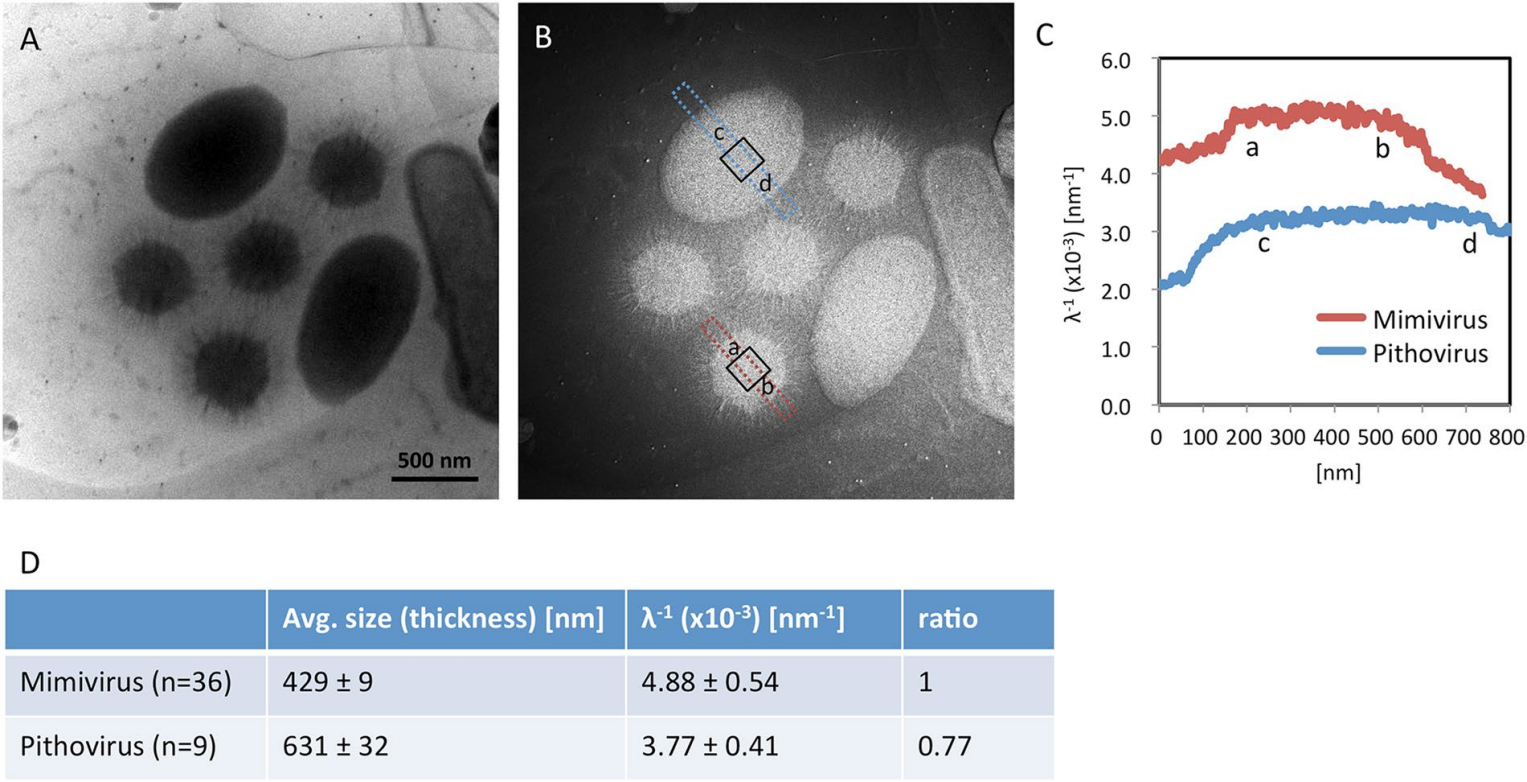
Measurement of nucleoid densities in Pithovirus and Mimivirus particles. (**A**) A bright field image without energy-filtering (cryo-EM at 200 kV). The small hexagonal and large oval shapes correspond to Mimivirus and Pithovirus particles, respectively. (**B**) Calculated EELS log-ratio image (t/λ, see Methods). Red and blue rectangles highlight regions for estimating nucleoid density profiles in Pithovirus and Mimivirus particles in (**C**). Black open boxes on the particles show the area used to statistically calculate the nucleoid density in (**D**). (**C**) Relative density line profiles (λ^−1^, see Methods) of Pithovirus and Mimivirus particles calculated from measurements in the red and blue bands in (**B**). The profile shows that the values of the central areas (a–b and c–d) on each viral particle reach a plateau, and the calculated density of Pithovirus was relatively lower than that of Mimivirus. (**D**) Statistics of the estimated relative nucleoid density of Pithovirus and Mimivirus particles. The relative nucleoid density (λ^−1^, see Methods) was calculated with each particle size (the particle width in Pithovirus; the capsid diameter in Mimivirus) in a central area of 150 nm × 150 nm and averaged in 9 Pithovirus and 36 Mimivirus particles. The nucleoid density of Pithovirus particle was estimated to be ~0.77 times the density of Mimivirus particles.

The nucleoid density of Pithovirus was compared to that of Mimivirus, using energy-filtered cryo-EM at 200 kV. In this experiment, Pithovirus and Mimivirus particles were mixed and imaged together in the same view with and without energy filtering (Fig. 8A). The calculated EELS log-ratio (t/λ) image (see Methods)^41^ is shown in Fig. 8B. In such large specimens like giant viruses, the viral particles protrude out of the ice surface; only a very thin layer of ice should cover the top of the specimens^21^. As a result, we can see that the ice thickness increases around the viral particles (Fig. 8B). Our electron tomography studies also showed that the viral particles did not suffer severe flattening in the ice film, where the ice thickness is appropriate for their size. Assuming that the sample thickness is close to the actual sample size, the interior density of the specimens can be estimated by dividing the EELS log-ratio (t/λ) image with each particle size (thickness t, see Methods)^42^. The resultant interior density profiles (λ^−1^) of Pithovirus and Mimivirus particles are shown in Fig. 8C. The profiles show that the values of the centre area (a-b and c-d in Fig. 8C) on each viral particle reach a plateau, and that the calculated density of Pithovirus is relatively lower than that of Mimivirus. The plateau densities (λ^−1^) of the central 150 × 150 nm area (black open boxes) on each viral particle were calculated for 9 Pithovirus and 36 Mimivirus particles, respectively, and averaged (Fig. 8D). The results show that the density of the nucleoid in Pithovirus particles is 0.77 times lower than the density of Mimivirus particles (Fig. 8D). Despite the size heterogeneity of the Pithovirus particles compared with the reproducible pseudo-icosahedral Mimivrus particles, the nucleoid density distribution in the Pithovirus particles is as uniform as that of Mimivirus particles (Fig. 8B,C). The nucleoid density of the Pithovirus particles has an 11% coefficient of variation, which is similar to the variation in the nucleoid density of the Mimivirus particles (Fig. 8D).

## Discussion

Several new families of unexpected viruses with large genomes and giant particles have been discovered since 2003^1,3,4,7,10^. However, ultra-structural studies at near native conditions of entire virus particles have so far been limited to the pseudo-icosahedral Mimivirus virion^11–13^ because of the specimen thickness. Here, we have attempted to investigate the entire structure of the largest ever-discovered virus particle of *Pithovirus sibericum*, at near-native conditions using electron cryo-microscopy and tomography.

We have identified an unexpected size variation of the Pithovirus particles. If we hypothesize that Pithovirus particles can pack a variable number of copies of the genome and the copy number determines the particle size, the size distribution would likely show a discontinuous profile. However, the size distribution does not show such multiple peaks (Fig. 1B). This suggests that the particle volume does not correlate with the genome copy number, and that the size distribution spans a continuum between about 900–2500 nm in length and 600–900 nm in width. The histogram also shows that there is no strong correlation between these two dimensions (Fig. 1B). We conclude that mature Pithovirus particles do not have a strictly enforced particle size, and their volumes do not show discontinuous clustering (Fig. 1C). Pithovirus particles are initially built as cylinders and then mature into their final amphora-like shape during the last stage in the assembly of the striated tegument in the host amoeba cell^7^. The size heterogeneity of the particles may reflect a natural flexibility of the virions. The rare Pithovirus particles displaying two opposite apical pores (Figs 1A and 2) are larger than the other particles and could be explained by fusion between two viral particles during the assembly process, likely impairing DNA loading^7^. Both phenomenon may be indicative of an unusual level of stochastic “noise” in the assembly of the Pithovirus particles.

Pithovirus particles appear cylindrical at low resolution and the viral capsid has no vertices (Fig. 5C,E). In this study, we observe that the virus particles are surrounded by a low-density outer layer (d in Fig. 3B). This low-density layer was not observed in chemically fixed and resin-embedded sections of the virus. The outer layer is about 40 nm thick and seems to be amorphous, and less dense than the Mimivirus protein fibres in images of cryo-frozen particles^13^. It could be speculated that the layer is made up from less dense biological materials such as polysaccharides. Mucus-like polysaccharide structures are difficult to observe in dried, fixed, and resin-embedded sections, but can be visualized in hydrated frozen specimens^43^.

It was previously shown that the apical cork, when viewed from the top, has a hexagonal honeycomb-like structure^7^. In the side view, the bundle structure of the cork shows perpendicular striations on the particle (Fig. 3C, black arrows). These stripes seem to be identical features of the apical cork that were observed earlier in chemically fixed and resin-embedded sections^7^. Under cryogenic condition, the apical cork shows parallel lines with a certain pitch on the perpendicular striations (Fig. 4, Supplementary Fig. S1). It could be due to the piling up of long coil-like fibres, showing a certain pitch in the side view, forming the vertices of the honeycomb structure. Further analyses of the cork are required to determine its detailed structure. It would be interesting to elucidate how the apical cork, a well-organized periodic structure, is assembled and inserted in the tegument, and whether the structures of the stripes in the tegument and the cork relate to each other.

The interior volume of the pseudo-icosahedral Mimivirus particle is about 1.4 × 10^7^ nm^3^ (~300 nm diameter) and harbours the 1181 kbp dsDNA genome inside the capsid^3^. In contrast, the interior volume of Pithovirus particles is 10–100 times larger (mean = 2.6 × 10^8^ nm^3^) than that of Mimivirus, but its dsDNA genome size is only 610 kbp, i.e. half of the Mimivirus genome. The estimated interior density of the Pithovirus particles, calculated from the EELS log-ratio images (Fig. 8), is approximately 3/4 of that of the Mimivirus particle. The tegument layer of the Pithovirus particles (TE in supplementary Fig. S2) and the capsid layer of the Mimivirus particles (CA in supplementary Fig. S3) are thought to be mainly composed of proteins. The relative nucleoid intensity of the Pithovirus particles is lower than that of the Mimivirus particles when they are standardized by the intensity of their respective outer protein layer (NU_intensity_/TE_intensity_ and NU_intensity_/CA_intensity_ in Supplementary Figs S2, S3). This agrees with the EELS result. Comparing their genomes and particle sizes, the Pithovirus genome should be ten to hundred times more sparsely packed than that of the Mimivirus. However, the interior density of the Pithovirus particle is calculated to be three quarters of that of the Mimivirus particle (Fig. 8). These results suggest that in addition to the genome, the interior of Pithovirus particles must be filled with other macromolecular compounds such as proteins, lipids, or non-genomic nucleic acids. The size variation is due to the plasticity of the particles and not likely due to the genome copy number. A more accurate assembly of such giant particles would probably require skeleton proteins to control their size and shape. Obviously, the lack of such tight control in particle formation did not significantly impact the fitness of Pithoviruses during their last 30,000 years of evolution, as indicated by the existence of a closely related contemporary relative^8^.

A previous study proposed that substructures might be present in the Pithovirus particles^7^, where the genome may be compartmentalized in the particle although this has not been demonstrated. Figure 6 shows membranous or densely organised structures in empty Pithovirus particles by energy-filtered cryo-EM at 200 kV. We note that such empty particles may have various origins such as assembly intermediates, defective particles or particles from which the dsDNA has been released. In the latter case, the membranous structures in the empty particles may correspond to the residual membrane after fusion between the virion and the host vacuole membrane or after releasing their DNAs accidentally. It is not clear at this point whether these structures are the intact interior sub-structures or post-processed structures, i.e. structures left behind following the release of the viral dsDNA. Figure 7 shows Zernike phase-contrast cryo-EM images of the DNA-filled Pithovirus particles. These images strongly suggest a compartmentalised interior of the intact particles. However, they show that the compartments are not visible in all particles and not uniform in each particle. It is also noted that the central hole of Zernike phase plate can filter the comparatively low-frequency scattered electron beam by changing the size of the hole. In a previous study of cyanobacterial cells, it was shown that the high-frequency information selectively enhances the contrast by the phase shift, whereby the interior fine structures of the cell are clearly observed^28^. This cut-on effect helps the observation of the internal membranous structures of the thick Pithovirus particles as well.

It was previously proposed that, in order to be propelled out and fuse with the phagosome membrane after the cork is expelled, the virion membrane needs to be folded^7^. Figure 5 shows that the root of the cork is sometimes occupied by a non-striated dense material (red area in Fig. 5D). It was also previously reported that there is a structure similar to a folded membrane in the region of this non-striated dense material^7^. Fusion between the two membranes could allow the transfer of the content of the nucleoid, including DNA and proteins, to the host cytoplasm. The Mimivirus particle has a dense area below the stargate portal and the portal seems to be important for releasing the genome^2,11,13,19^. PBCV-1 has a pocket underneath the unique 5-fold vertex and the pocket is thought to pack the enzymes needed to digest the host cell wall^44^. In a similar manner, the dense material underneath the unique cork of Pithovirus particles may contain critical structures or complexes for releasing the genome and infecting the host. However, details of these processes are difficult to observe in these thick and densely filled particles. The present cryo-EM studies show an alternative way to characterize structural details of the giant viruses.

## Methods

### Sample purification and specimen preparation for cryo-EM

Pithovirus and Mimivirus were purified as described previously^3,7^. Viral samples were mixed with the same amount of 15 nm BSA-conjugated colloidal gold (AURION, The Netherlands) as fiducial markers for tomographic alignment. Approximately 3 µL of the samples were applied on R1.2/1.3 or R3.5/1 Quantifoil grids (Quantifoil Micro Tools GmbH, Germany) glow-discharged beforehand, and plunged-frozen in liquid ethane using a Vitrobot Mark IV (FEI Company, USA).

### Energy filtered cryo-EM

The frozen grids were mounted on a 914 liquid-nitrogen cryo-specimen holder (Gatan Inc., USA) and loaded into a 200 kV field-emission electron microscope equipped with an omega-type in-column energy filter with 10 eV slit width (JEM2200FS, JEOL, Japan). Raw images were recorded on a 4 K × 4 K charge-coupling device sensor (F415, TVPIS, Germany) with an acceleration voltage of 200 kV using a low-dose mode. The contrast-enhanced images were taken with a Zernike phase plate^25,26^.

### High-voltage Cryo-EM and electron tomography

Frozen grids were mounted on the liquid-nitrogen cooled cryo-specimen holder (Gatan Inc., USA) and loaded into the Hitachi H-1250M high voltage electron microscope (Hitachi Hi-Tech Fielding Corp., Japan). Raw images were recorded with an acceleration voltage of 1 MV on a 2 K × 2 K CCD sensor at the nominal magnification of ×10,000 using a low dose condition. 41 tilted images of Pithovirus particles at the range from −60 to 60 degrees with a tilting step of 3 degrees were manually collected with underfocuses of 5 to 10 μm and a total dose less than 100 e^−^/Å^2^ for tomographic 3D reconstruction. The original images were collected with 18.75 Å /pixel on the CCD. The images were aligned and binned by 2 for generating a final 3D model using the IMOD software^45^ without CTF correction. The 3D reconstruction was performed using the SIRT algorithm. The attainable image resolution was estimated to be less than 75 Å. The 3D models of the Pithovirus particles were segmented and coloured by the Amira software (FEI Visualization Sciences Group, USA).

### Density measurement of Pithovirus and Mimivirus particles

The difference of nucleoid density between Pithovirus and Mimivirus was accessed by using energy-filtered cryo-EM images. Pithovirus and Mimivirus particles were mixed and embedded in vitreous ice on a R3.5/1 Quantifoil grid using the same procedure as mentioned earlier. The frozen grid was loaded into the 200 kV energy-filtered cryo-EM, JEM2200FS using a Gatan 914 cryo-specimen holder. The images of the viruses were collected at the same area on the 4k × 4k CCD camera with and without the energy slit of 10 eV. The objective aperture was removed to reduce an electron scattering error^21^. The two images were taken with an electron dose of ~15 e^−^/Å^−2^ each on the same specimen area to avoid specimen damage. Then, EELS log-ratio images^41^ were calculated with and without energy filter images, according to formula (1),

1 $$\frac{t}{\lambda }=\,\mathrm{ln}\,\frac{{I}_{tot}}{{I}_{0}}$$

where t is the specimen thickness, λ the inelastic mean free path, I_tot_ the total integrated intensity (unfiltered image intensity), and I_0_ the integrated intensity of the zero-loss peak (filtered image intensity). In the case of the giant viruses, the ice thickness increases around the viral particles (Fig. 8A and B), where the viral particles protrude out of the ice surface; only a very thin layer of ice should cover the particles^21^. In addition, since our electron tomography observations showed that the viral particles did not suffer severe flattening in the covering thin ice film, we can assume that the ice thickness is appropriate for their particle size (width). Therefore, the relative density of the two viruses was estimated by comparing with each value of λ^−1^, which were divided with the EELS log-ratio image value of each sample thickness (t). λ is described by formula (2),

2 $$\lambda ={[{\sum }_{i}{n}_{i}\sigma ]}^{-1}$$

where n_i_ is the cross section per molecule, σ_i_ the number of molecules per unit volume, i different types of molecules in the sample^42^. Consequently, the calculated intensities of λ^−1^ of the viral particles can be used to compare the interior density of the two viruses. The relative nucleoid density is calculated in a central area of 150 × 150 nm and averaged in 9 Pithovirus and 36 Mimivirus particles.

## Electronic supplementary material

Supplementary movie 1

Supplementary movie 2

Supplementary movie 3

Supplementary figures S1, S2, S3
