# Supplementary material for: Structural variability and complexity of the giant Pithovirus sibericum particle revealed by high-voltage electron cryo-tomography and energy-filtered electron cryo-microscopy
Source: Sci Rep. 2017 Oct 16;7:13291. doi: 10.1038/s41598-017-13390-4 (PMC5643343; doi:10.1038/s41598-017-13390-4)
Supplement: Supplementary file 4 — Supplementary figures S1, S2, S3 [file 41598_2017_13390_MOESM4_ESM.pdf]

Structural variability and complexity of the giant *Pithovirus sibericum* particle revealed by high-voltage electron cryo-tomography and energy-filtered electron cryo-microscopy

Kenta Okamoto[1]*, Naoyuki Miyazaki[2], Chihong Song[2], Filipe R.N.C. Maia[1], Hemanth K. N. Reddy[1], Chantal Abergel[3], Jean-Michel Claverie[3,4], Janos Hajdu[1,5], Martin Svenda[1], and Kazuyoshi Murata[2]*

1. Laboratory of Molecular Biophysics, Department of Cell and Molecular Biology, Uppsala University, Husargatan 3 (Box 596), SE-75124 Uppsala, Sweden
2. National Institute for Physiological Sciences (NIPS), Okazaki, Aichi, 444-8585 Japan
3. Structural and Genomic Information Laboratory, UMR 7256 (IMM FR 3479) Centre National de la Recherche Scientifique & Aix-Marseille University, Marseille, 13288, France
4. Assistance Publique des Hôpitaux de Marseille. La Timone, 13005 Marseille, France.
5. Institute of Physics AS CR, v.v.i., Na Slovance 2, 18221 Prague 8, Czech Republic

Running title: High-voltage cryo-tomography of Pithovirus

*Correspondence to KO (kenta.okamoto@icm.uu.se) and KM (kazum@nips.ac.jp)

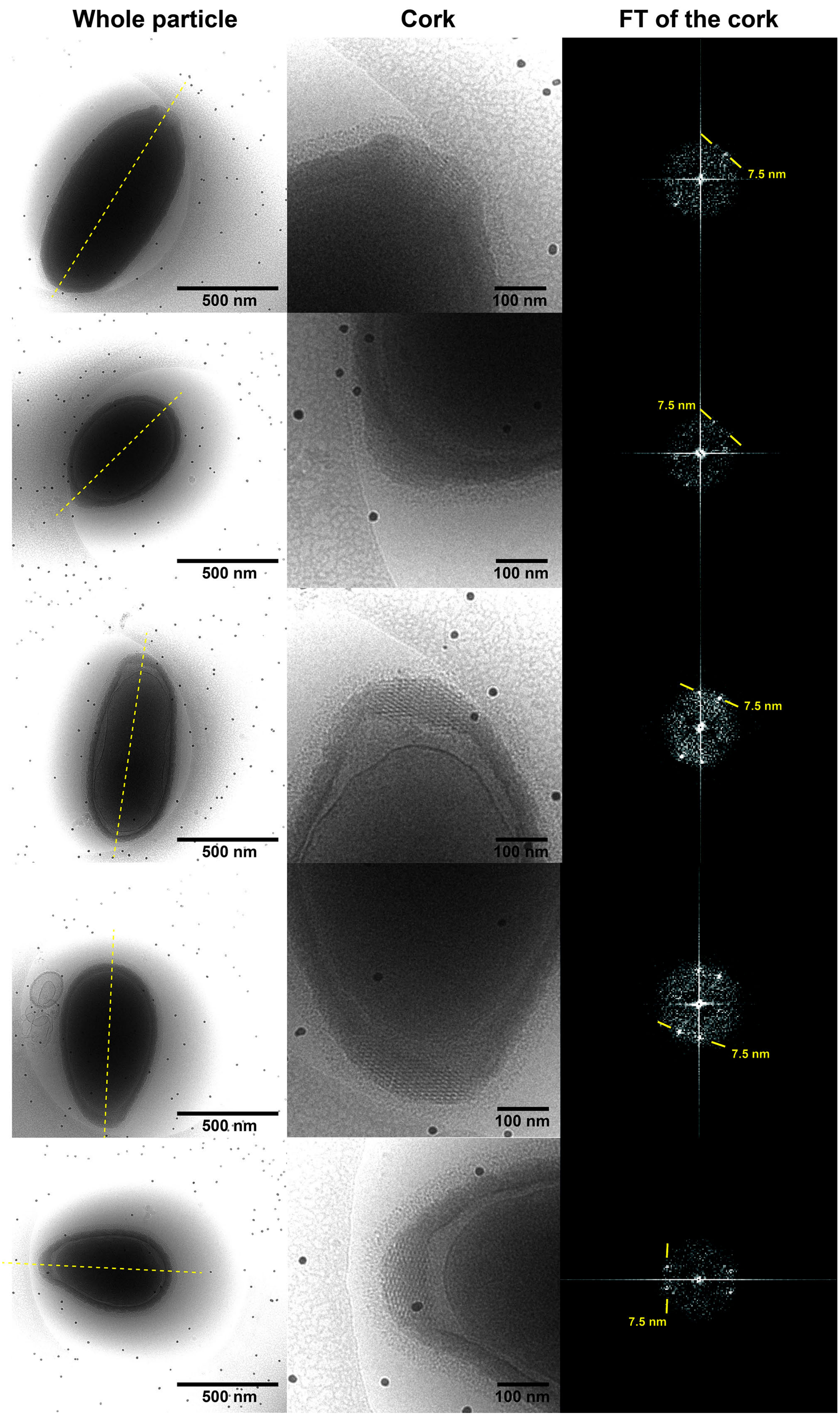

Whole particle
Cork
FT of the cork
500 nm
100 nm
7.5 nm
500 nm
100 nm
7.5 nm
500 nm
100 nm
7.5 nm
500 nm
100 nm
7.5 nm
500 nm
100 nm
7.5 nm

Supplementary Fig S1 Periodic Moiré patterns of the apical corks in side view of five Pithovirus particles using energy-filtered cryo-EM at 200 kV. Whole particle (left), the cork (middle) and the Fourier transform of the cork (right). The dotted yellow line in the whole particles show the central line of the particles along the major axis. The yellow lines in the right panels indicate the pitch of the Moiré pattern on the cork. The pitch of the parallelly aligned lines is 7.5 nm in all the particles lying sideways. The constant spacing could be because the particles aligned in the vitreous ice and the alignment was limited in the vertical direction.

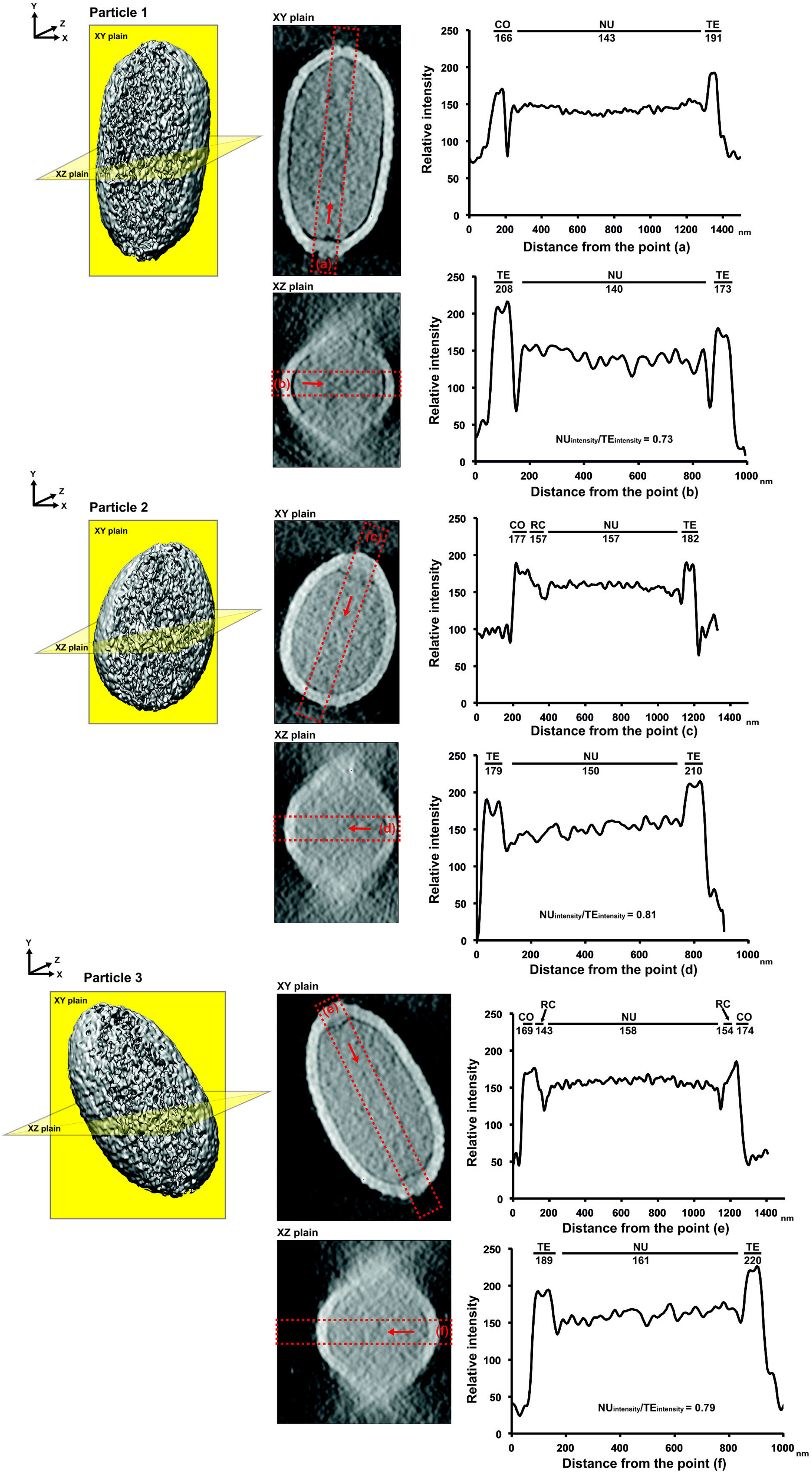

Particle 1
XY plain
XZ plain
Y
Z
X
CO 166
NU 143
TE 191
Relative intensity
Distance from the point (a)
nm
TE 208
NU 140
TE 173
NUintensity/TEintensity = 0.73
Distance from the point (b)
Particle 2
CO 177
RC 157
NU 157
TE 182
Distance from the point (c)
TE 179
NU 150
TE 210
NUintensity/TEintensity = 0.81
Distance from the point (d)
Particle 3
CO 169
RC 143
NU 158
RC 154
CO 174
Distance from the point (e)
TE 189
NU 161
TE 220
NUintensity/TEintensity = 0.79
Distance from the point (f)

Supplementary Fig. S2 Relative intensity profiles across the Pithovirus particles in the XY and XZ plans of the 3D tomograms. Three different sizes of the particles (Particle 1 – 3) in Fig. 5B are analyzed. The histograms of the relative intensity profiles from the point (a) – (f) are shown (right). The 150 nm thick lines (red dotted area) are used for calculating the relative intensity. The slices are divided into the assigned regions of the cork (CO), the nucleoid (NU), the tegument (TE), and the root of the cork (RC). The averaged relative intensities of them are shown on the X-axis. The standardized averaged nucleoid intensity was calculated by dividing the averaged relative intensity with the tegument intensity ($NU_{intensity}/TE_{intensity}$).

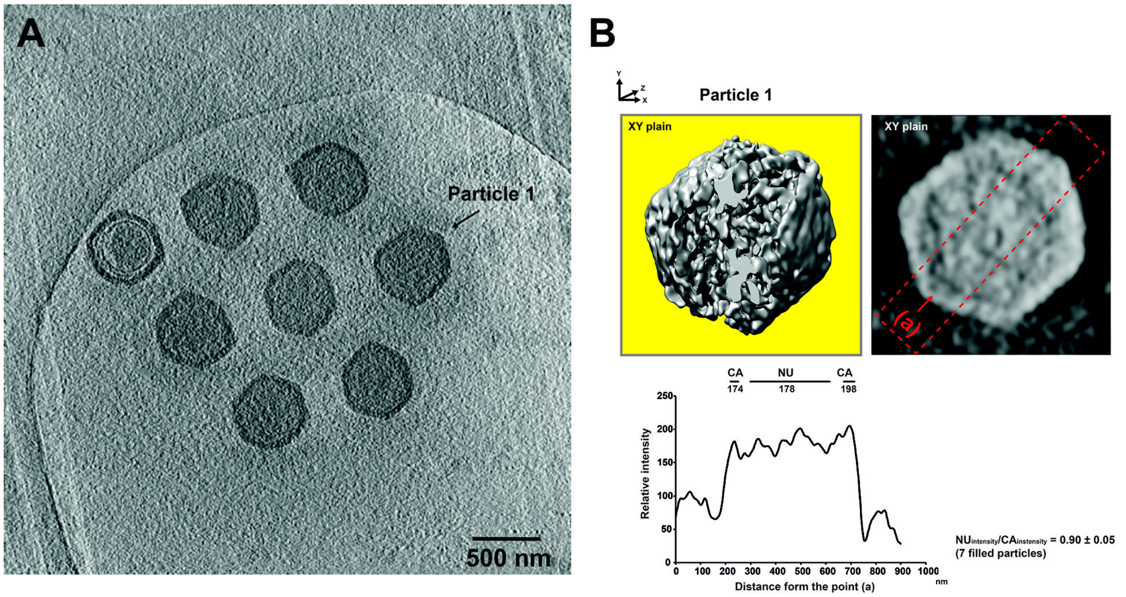

Supplementary Fig. S3 Tomographic 3D reconstruction of Mimivirus particles using cryo-HVEM at 1 MV. A) 3D tomogram of Mimivrus particles. B) Relative intensity across the representative Mimivrus particle in the XY plan of the 3D tomogram. The histogram of the relative intensity profile from the point (a) is shown (bottom). The 150 nm thick line (red dotted area) is used for calculating the relative intensity. The slice is divided into the assigned regions of the capsid (CA) and the nucleoid (NU). The averaged relative intensities of them are shown on the X-axis. The standardized averaged nucleoid intensity was calculated by dividing the averaged relative intensity with the tegument intensity ($NU_{intensity}/CA_{intensity}$) and averaged in 7 Mimivirus particles.
